# A combined microinvasive trans-submandibular and nasendoscopy surgical approach to dissect recurrent or radiotherapy-insensitive nasopharyngeal carcinoma

**DOI:** 10.3389/fonc.2022.939404

**Published:** 2022-08-17

**Authors:** Yanming Zhao, Jugao Fang, Qi Zhong, Luo Zhang, Chengsuo Wang, Jiamin Zhang, Jiaming Chen, Ling Feng, Shizhi He, Hongzhi Ma, Lizhen Hou, Meng Lian, Qian Shi, Xixi Shen, Yifan Yang, Ru Wang

**Affiliations:** Department of Otorhinolaryngology Head and Neck Surgery, Beijing Tongren Hospital, Capital Medical University, Beijing, China

**Keywords:** parapharyngeal space, nasopharyngeal carcinoma, surgerical procedures, nasendoscopes, combined

## Abstract

**Objective:**

To investigate a novel combined microinvasive trans-submandibular and nasendoscopy surgical approach for nasopharyngeal carcinoma involving the parapharyngeal space.

**Methods:**

Seven patients diagnosed with nasopharyngeal carcinoma involving the parapharyngeal space between May 2018 and April 2021, two males and five females, aged 37–63 years.Six of the 7 patients underwent submental flap preparation and dissection of the lymph nodes in the upper neck and parapharyngeal space on the lesion side. The nasopharynx lesions and tumor margins were dissected under nasal endoscopy. The medial boundary of internal carotid artery separated by open cervical approach was used as the lateral boundary of the tumor to realize en bloc resection of the tumor.

**Results:**

The patients were preoperatively diagnosed with T2~3N0M0 nasopharyngeal carcinoma, including mucoepidermoid carcinoma (n=2), papillary adenocarcinoma (n=1), and nonkeratinizing squamous cell carcinoma (n=4). The tumors were removed completely, and patients achieved primary healing of the incision. No recurrence and no serious complications were recorded during the 13–48 month follow-up.

**Conclusion:**

Complete resection of the tumor was obtained in the 7 patients without recurrence and serious complications during the follow-up. The findings of this cohort study suggest that, patients with recurrent nasopharyngeal carcinoma after radiotherapy and radiotherapy-insensitive types of nasopharyngeal carcinoma, the combined microinvasive trans-submandibular and nasendoscopy surgical approach may be considered as an surgical options. The results of this study provide an additional option for surgical treatment of NPC in the clinic.

## Introduction

Surgery has become the treatment of choice for recurring nasopharyngeal adenocarcinoma (NPC) after radiotherapy, and some special tumor types insensitive to radiotherapy, such as adenoid cystic carcinoma, mucoepidermoid carcinoma, and nasopharyngeal adenocarcinoma (1). However, treatment of NPCs that involve the parapharyngeal space (PPS) is challenging due to their position deep in the pharynx. Besides intractable bony structures around the nasopharynx, the region includes important structures such as the internal carotid artery, cavernous sinus, and optic nerve. Dissection difficulties during surgery could lead to disastrous consequences if any of these is damaged. It is difficult to expose the parapharyngeal segment of the internal carotid artery by endoscopy alone, making it difficult to completely resect NPC tumors that invade or border the PPS due to the elevated risk of damaging the internal carotid artery. Consequently, tumor residues occasionally remain, increasing the risk for recurrence.

Various approaches, including infratemporal, transcervico-mandibulo-palatal, transpalatal, transmaxillary, transcervical, maxillary swing, facial translocation, and degloving, have been developed to dissect tumors radically (2). However, these open surgical methods usually involve the destruction of bone structures, cause facial scarring, impair facial aesthetics, and might result in postoperative osteonecrosis that could seriously affect the patients’ quality of life. It is a great challenge for surgeons to perform such operations to completely remove the tumor while protecting anatomical structures such as the internal carotid artery and facilitating wound repair.

We attempted a novel tumor resection surgical procedure that enables safe and thorough dissection of the PPS, including the internal carotid artery segment, and performed nasopharyngeal wound repair using a cervical-parotid approach. The procedure includes the following steps: a) create a submental flap; b) dissect the internal carotid artery through a cervical-parotid approach to the PPS; c) dissect the lateral border of the tumor; d) dissect the nasopharynx by an endoscopic approach. The complete resection of the tumor is achieved by a combination of an “inside and outside” approach. The presented method is convenient and excellent in protecting the internal carotid artery while completely removing the tumor and performing nasopharyngeal wound repair.

This study aimed to analyze the advantages and limitations of a combined trans-submandibular and endoscopic surgical approach to the PPS by reporting the outcomes of a series of patients with recurrent or radiotherapy-insensitive NPC.

## Patients and methods

### Study population

This retrospective study included seven patients (two male and five female), aged 51.57 ± 7.52 (range, 37–63) years, diagnosed with NPC at the Department of Otolaryngology-Head and Neck Surgery of Beijing Tongren Hospital, affiliated with Capital Medical University, Beijing, between May 2018 and April 2021. Surgical resection was recommended by comprehensive multidisciplinary discussion in all cases. The clinical research ethics committee of Beijing Tongren Hospital approved this study. The characteristics of our study population are shown in **Table 1**.

**Table 1:** Patient characteristics.

cases	Age (years)	Sex	Smoking history	Drinking status	Histotype	T, N, M stage	Adjuvant therapy	Complications	Follow-up (months)
1	56	Male	Yes	Yes	rNPC	T4N2M0	Chemotherapy	Facial sweating, pain when lifting the right hand	48
2	49	Female	No	No	MEC	T2N0M0	None	None	41
3	48	Female	No	No	PA	T3N0M0	None	None	33
4	55	Male	Yes	No	rNPC	T2N0M0	None	None	31
5	37	Female	No	No	rNPC	T2N2M0	None	Diplopia	28
6	63	Female	No	No	MEC	T4N0M0	IMRT	Xerostomia	20
7	53	Female	No	No	rNPC	T4N2M0	None	Nasal blockage, choking on eating	13

rNPC, recurrent nasopharyngeal carcinoma; MEC, mucoepidermoid carcinoma; PA, papillary adenocarcinoma; IMRT, intensity-modulated radiotherapy.

### Surgical technique

Six of the seven patients were treated with a submental island flap for reconstruction, and lymph nodes in the upper neck and PPS on the lesion side were dissected. The vascular sheath of the PPS at the base of the skull was dissected superiorly through a cervical-parotid approach and medially from the PPS to the nasopharynx. The tumor was resected using a combination of internal and external approaches. The nasopharynx lesions and tumor margins were dissected under nasal endoscopy. The medial boundary of internal carotid artery separated by open cervical approach was used as the lateral boundary of the tumor to realize en bloc resection of the tumor.

**Figure 1:**
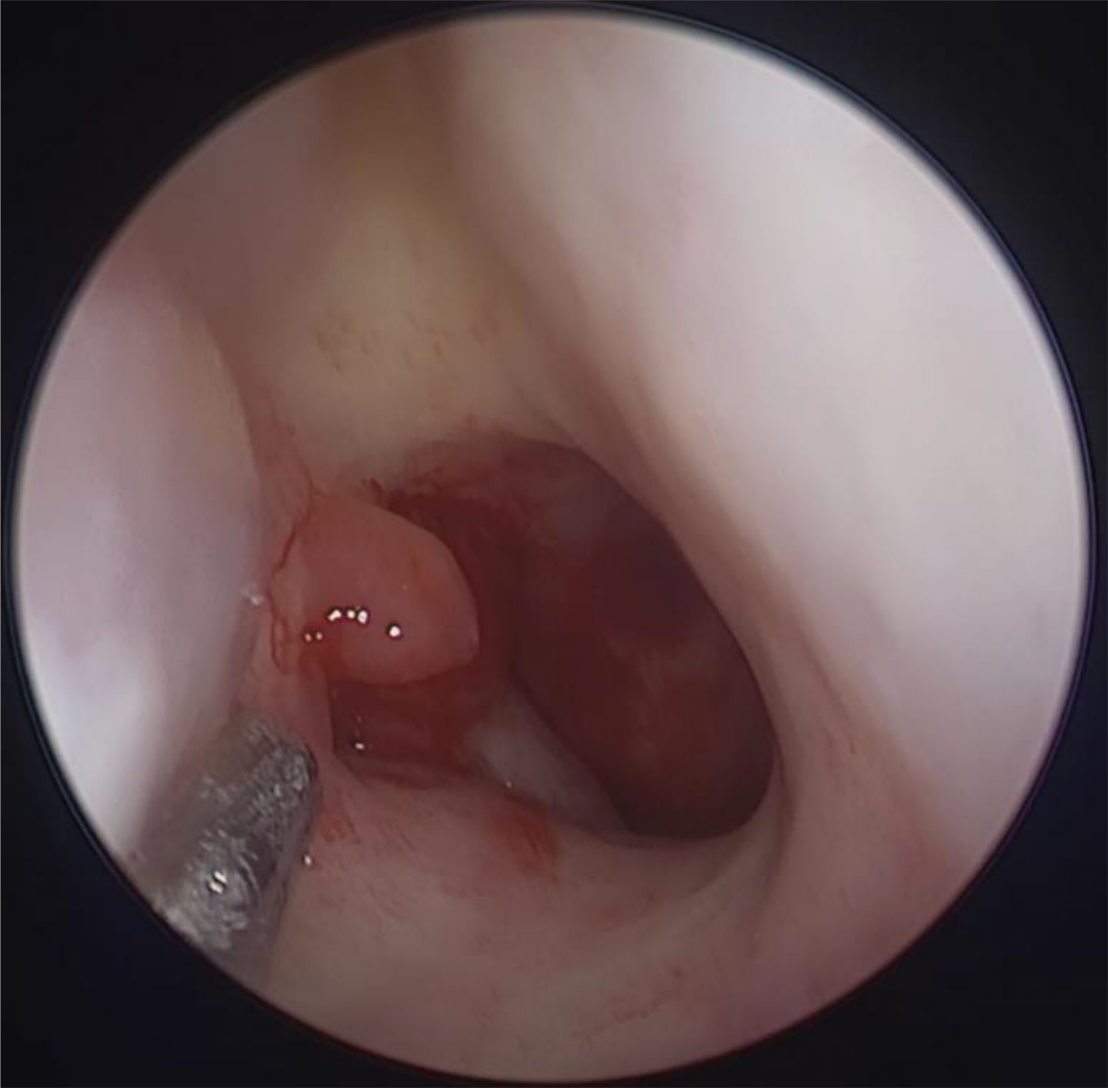
Nasopharynx tumors under nasal endoscopy: Nasoendoscopy revealed a left-sided nasopharyngeal mass.

**Figure 2:**
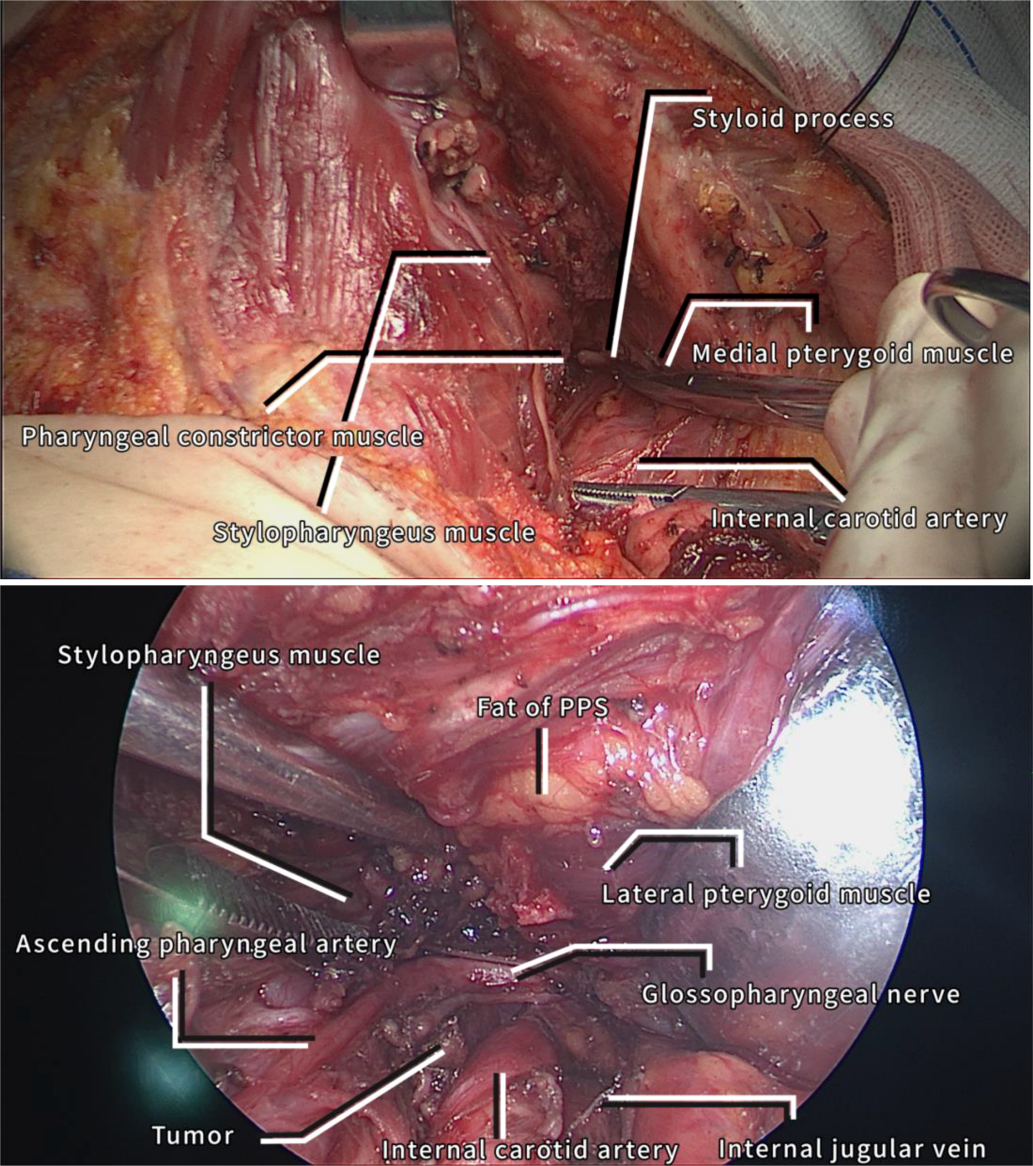
The submental flap is made complete and exposes the PPS and PPS under nasoendoscopic view.

**Figure 3:**
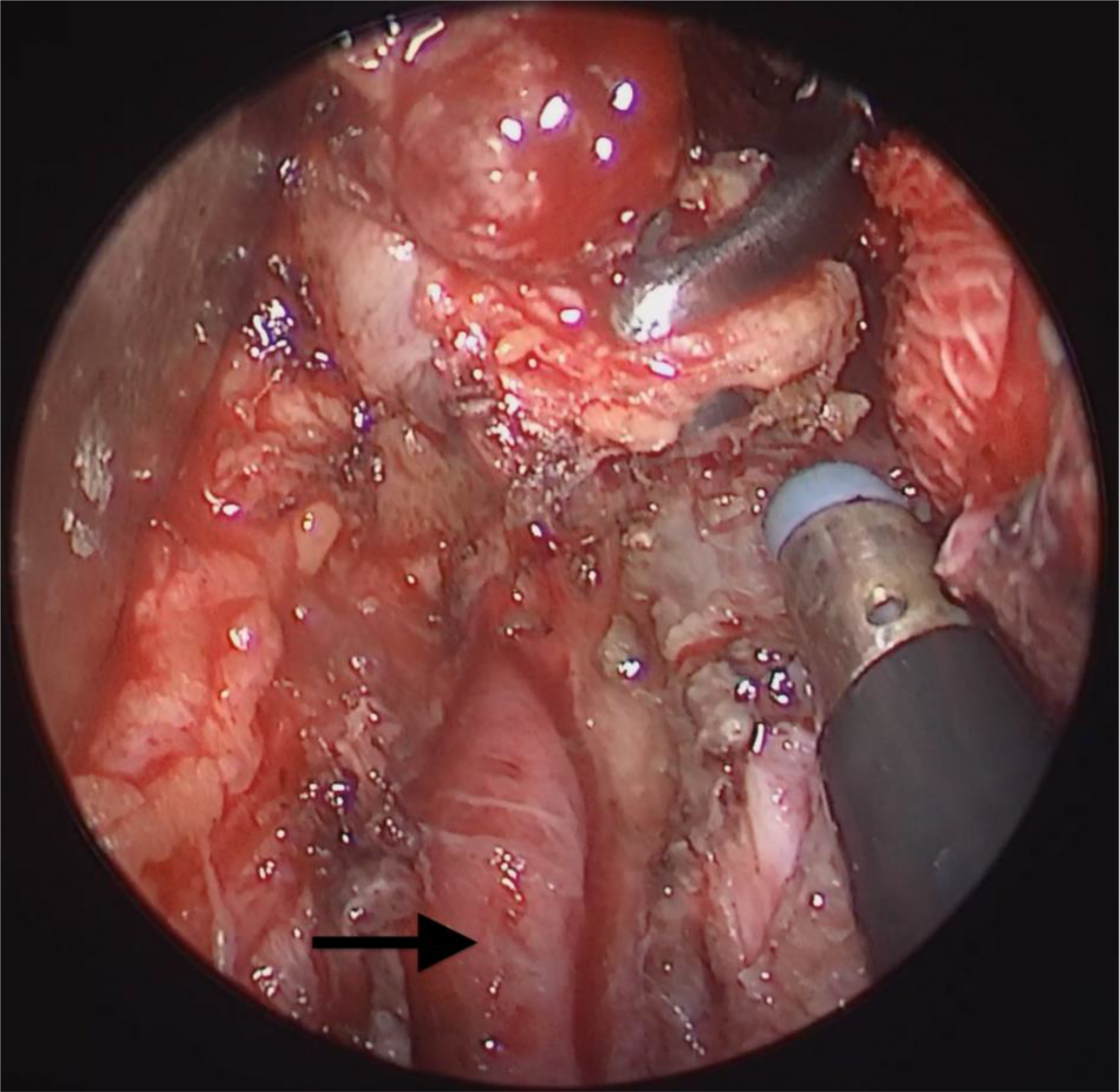
Nasal endoscopy combined with external cervical approach to expose tumors: Exposure of the internal carotid artery and tumor resection with safe margins by combined nasal and submandibular approaches. Ethmoid forceps in the nasal approach pulled the mass medially. Arrow: parapharyngeal internal carotid artery.

**Figure 4:**
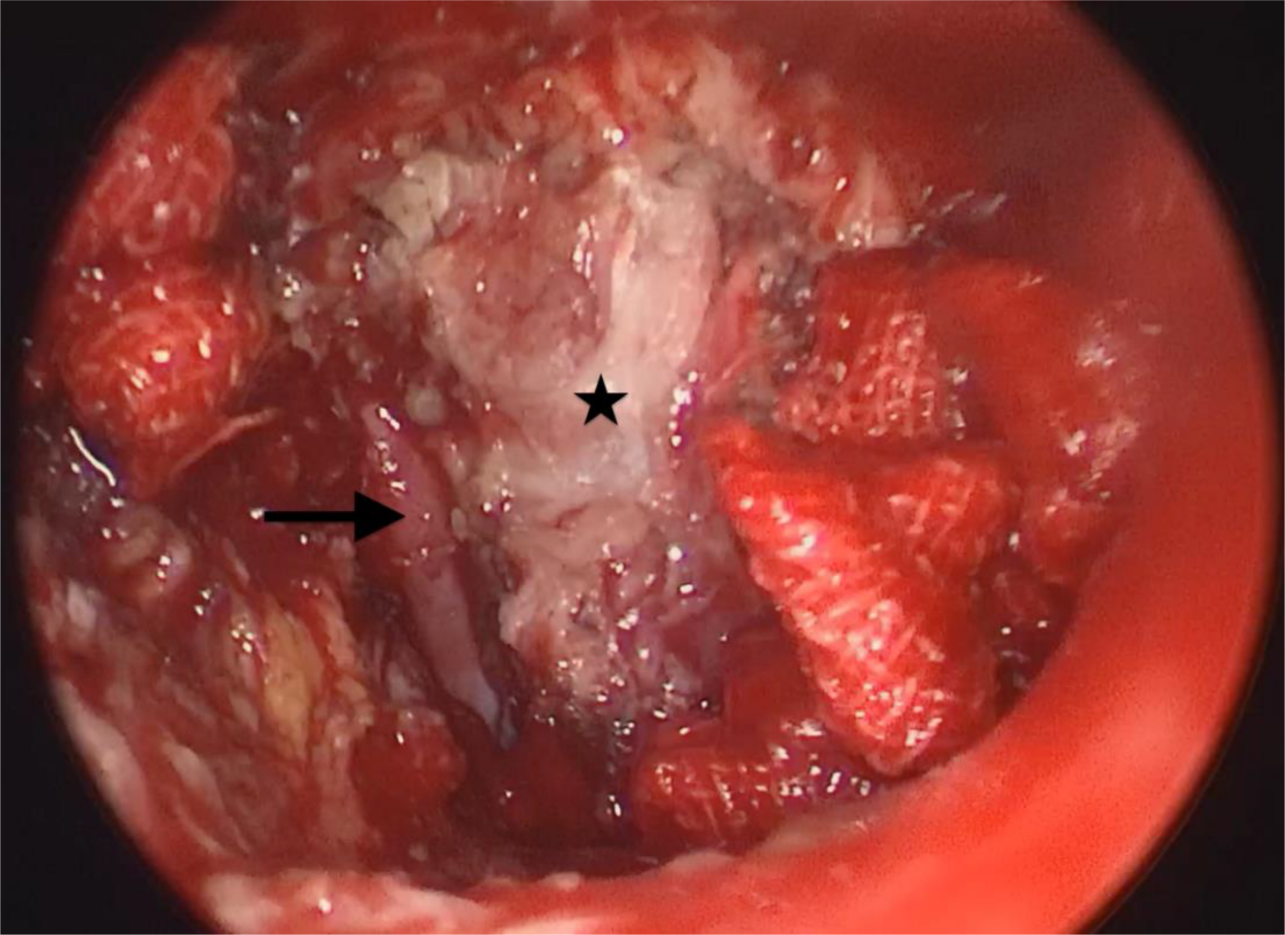
Postoperative field status after tumor resection. The parapharyngeal internal carotid artery dissected through an external cervical approach was visualized by nasendoscopy. Arrow: parapharyngeal internal carotid artery; star: right posterior pharyngeal wall after resection of the nasopharyngeal tumor.

**Figure 5:**
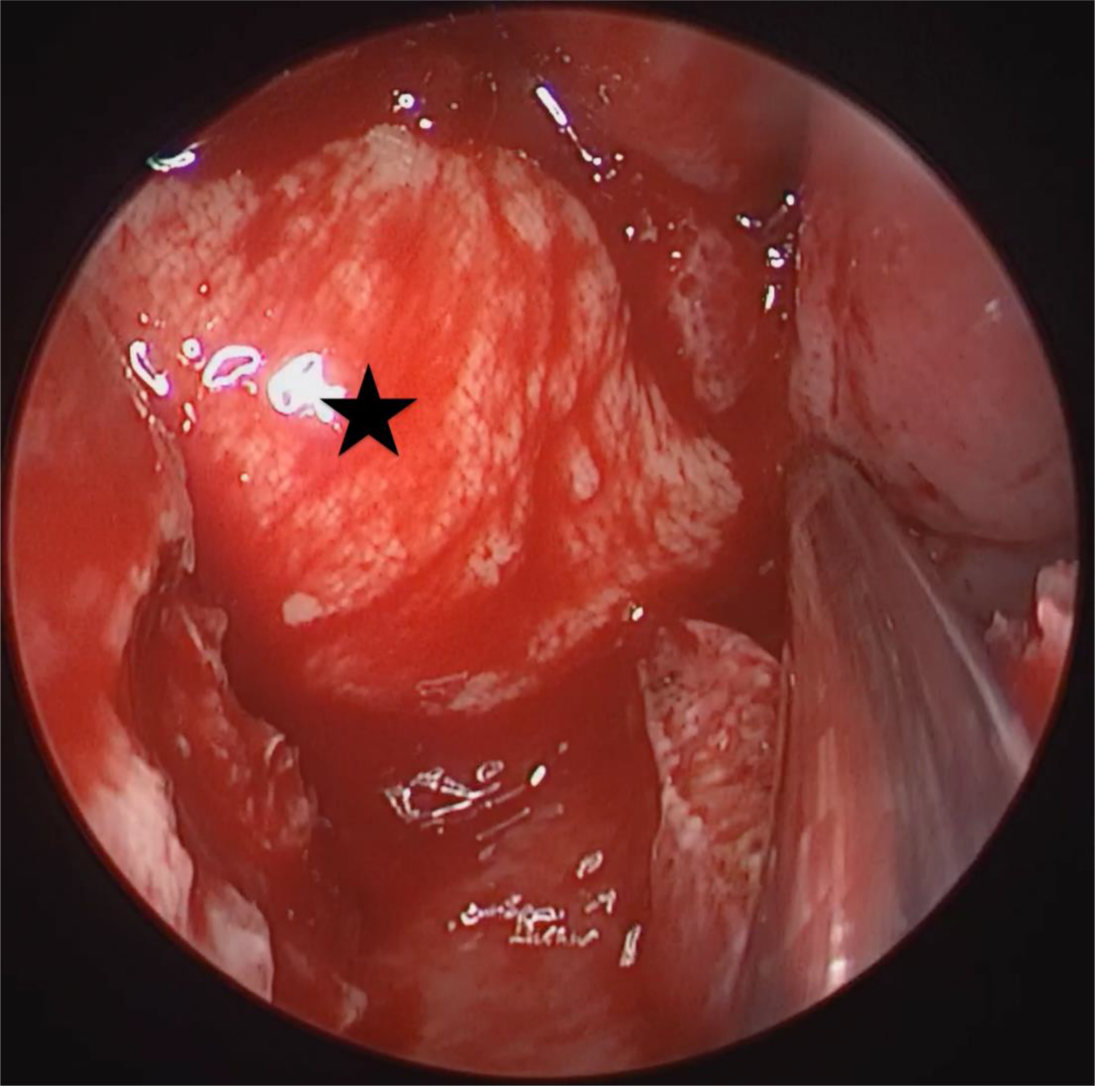
Repair of wound with submental flap: A submental flap was passed through to cover the internal carotid artery in the parapharyngeal space and repair the nasopharynx wound. Star: submental flap.

**Figure 6:**
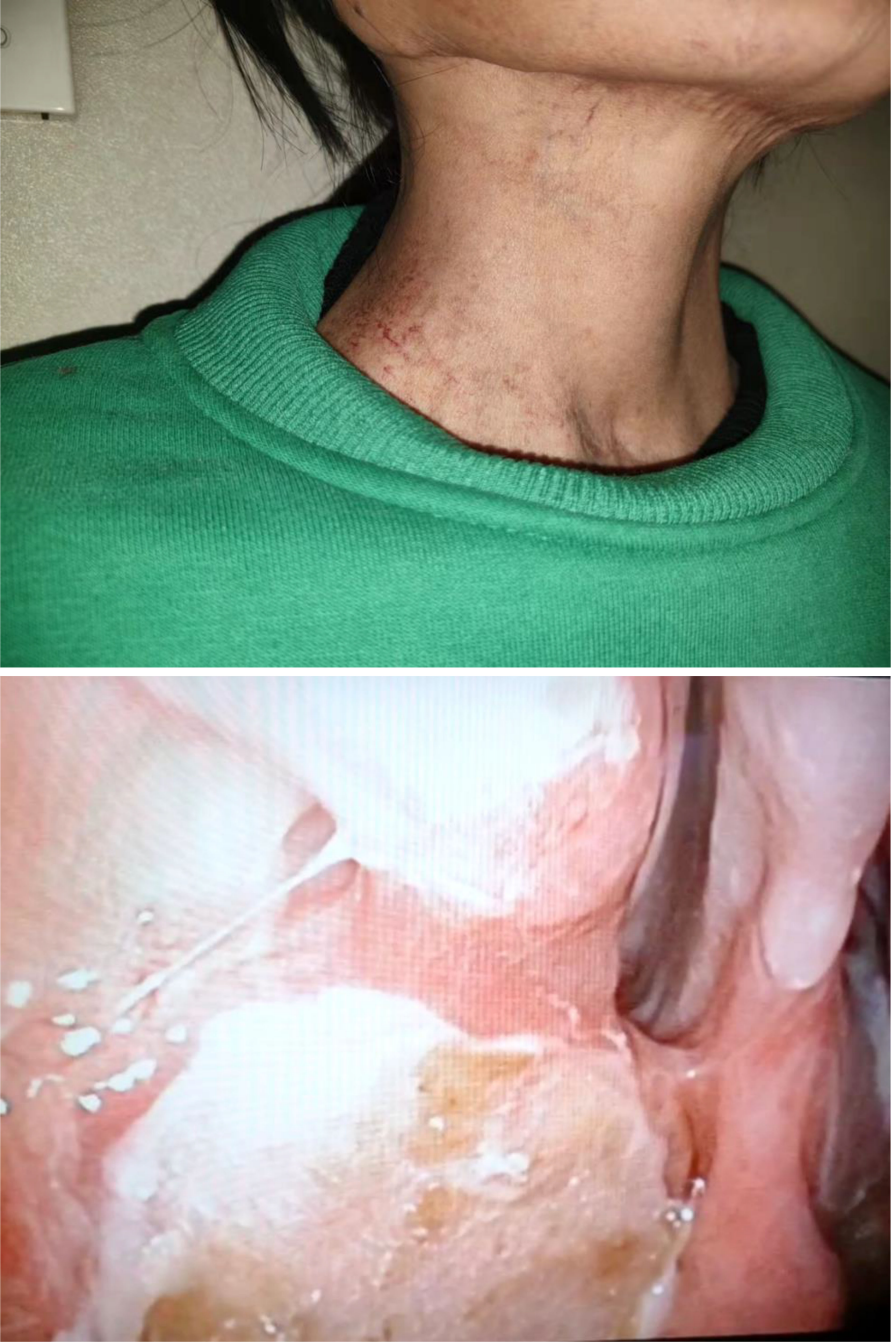
Postoperative follow-up photographs: When reviewed ten months postoperatively, the submental scar was invisible, and the repair with the submental flap in the nasopharynx was excellent.

The specific surgical procedure was as follows: A transverse incision was made at 1 cm from the lower border of the mandible. The marginal manibular branch of the facial nerve was protected during the dissection, the starting point of the submental artery was detected, and a submental perforator flap was made by dissection toward the midline of the neck.The digastric muscle was cut at the middle bond when making the submental flap, the anterior belly of the digastric muscle and part of the mylohyoid muscle were cut together with the submandibular gland flipped outward to form the vascular pedicle structure of the submental flap. The stylohyoid muscle and the posterior belly of the digastric muscle were dissected poster laterally. Take care to protect the hypoglossal nerve. The carotid sheath was dissected upward along the internal jugular vein, the internal carotid artery was dissected all the way from the anterior to the medial aspect of the carotid sheath, until near the rupture hole. During dissection, the stylohyoid and stylopharyngeal muscles are cut, the medial pterygoid muscles are exposed first, and the glossopharyngeal nerve is dissected superiorly and protected, at which point the stylohyoid process can be fully exposed, and a yellow PPS above the stylohyoid process is visible containing fat and lymphoid tissue. The fat and lymphoid tissues in the PPS were excised and the lower border of the lateral pterygoid muscle was bare. The medial pterygoid muscles, lateral pterygoid muscle were dissected with a bipolar or a harmonic scalpel, and tumors that protruded into the PPS were located superiorly medial to the internal carotid artery and lateral to the pharyngeal superior constrictor muscle. The perpendicular plate and pterygoid bone were grinded under nasal endoscopy. Removed the sphenoid sinus ostium and sphenoid septum, dissected the contralateral safety boundary of the tumor in the nasopharynx, dissected the tumor from the surface of the longus capitis muscle, beneath the sphenoid bone, and removed the tumor from the oral or PPS. After resection of the tumor, a natural passage through which the submental flap can be pulled upward into the nasopharynx to repair the wound in the denuded internal carotid artery, medial cavernous sinus, and posterior nasopharyngeal wall is created. Unless no cervical lymph nodes remained, we routinely dissected lymph nodes of Levels IB, II, and Va. In cased of metastases, we handled them following the neck lymph node dissection principles. We carefully protected the glossopharyngeal, hypoglossal, accessory, and vagal nerves. We made sure not to mistaken the superior sympathetic ganglion on the deep surface of the internal carotid artery for a lymph node and perform lymphadenectomy. After adequate wound irrigation, the premade submental flap was moved over the PPS to the nasopharynx to repair the defects and cover the wounds. Subsequently, a nasopharyngeal iodoform yarn strip was packed and placed, and a negative pressure drainage tube was placed in the PPS. Finally, the neck incision was closed routinely. **Figures 1**–**6** show some key steps in the surgical process.

### Postoperative management

The cervical PPS drainage tube was removed 2–3 days after the operation. The iodoform yarn stripe was removed from the nasal cavity 7–8 days postoperatively, followed by daily nasal irrigation. The patient visited the outpatient clinic 1–2 weeks postoperatively for review and nasal clearance. Patients requiring radiotherapy after the surgery started treatment 4–5 weeks postoperatively. Nasal feeding was usually maintained for 5–7 days.

### Adjuvant treatment

We followed the multidisciplinary comprehensive therapy (MDT) pattern and chose a rational use of radiotherapy, chemotherapy, immunotherapy, or other means for recurrent and radiotherapy-insensitive NPC postoperatively. We aimed to develop an individualized comprehensive treatment strategy when possible to improve efficacy while ensuring the patient’s survival and quality of life.

## Results

### Postoperative outcomes

The study included seven patients treated by the combined trans-submandibular PPS and endoscopic surgical approach. No major surgical complications occurred. Minor complications observed included facial sweating and pain in lifting the right hand, diplopia, xerostomia, and nasal blockage with choking on eating, each in one patient (**Table 1**).

### Pathological findings

Among the seven patients, four had nonkeratinizing, undifferentiated recurrent NPC after radiotherapy, two were with mucoepidermoid carcinoma of the nasopharynx (one poorly differentiated, one of low grade), one was with nonkeratinizing papillary adenocarcinoma. Two patients with recurrent NPC were confirmed to have cervical lymph node metastases, one with bilateral metastases (all at Level II-III; left, 1/5; right, 4/9) and the other with unilateral metastases (left; Level II-III, 2/8 and Level I, 2/3). Rapid intraoperative tumor margins pathologic results were negative in all patients.

### Survival outcomes

The mean follow-up time was 31(median, 31; range 13–48) months. None of the patients died from the disease during follow-up, and no recurrences were observed. While the patients had slight paresis of the facial nerve mandibular ramus postoperatively, all recovered completely within three months.Postoperatively, there was only a short period of small crusting in the nasopharynx incision that had disappeared within 2–3 months. The patients had good nasal ventilation and no significant decrease in nasal function.

## Discussion

Nasopharyngeal carcinoma is a global disease, but it shows marked geographical differences, with a high incidence of approximately 25–50/100,000 in China and Southeast Asian countries (3), and under 1/100,000 in Europe, the American continent, and Oceania (4). At present, the accepted and effective radical treatment for NPC is radiotherapy or radiotherapy-based comprehensive treatment.

Although intensity-modulated radiotherapy has significantly decreased the NPC local recurrence rate, 10–15% of patients still experience recurrence (5–7). The MDT pattern should be followed for recurrent NPC, with the present treatment being primarily re-radiotherapy and salvage surgery. However, it has been reported that approximately 12–28% of patients managed with radiotherapy for recurrent NPC had severe complications such as radionecrosis, temporal lobe necrosis, massive hemorrhage from a ruptured internal carotid artery, and cranial nerve dysfunction (8). Salvage surgery avoids the serious complications of re-radiotherapy but is comparable to it in local control and survival rates and guarantees the patient’s quality of life. Following its recent improved efficacy, the approach has gained wide acceptance among scholars (1). Besides, a very small proportion of malignant tumors originating from the minor salivary glands of the nasopharynx, such as adenocarcinomas, adenoid cystic carcinomas, and mucoepidermoid carcinomas, are insensitive to radiotherapy and require surgery followed by adjuvant radiotherapy.

Because of their deep location and numerous surrounding vital structures in the nasopharynx, it is extremely difficult to completely remove tumors from this region while protecting the surrounding vital tissues. The traditional open surgical approaches, such as transhiatal resection through a nasolabial incision, transhiatal resection via a maxillofacial approach, and maxillary swing resection via a lateral cervical approach, are aggressive and effective treatments for recurrent NPC, with 5-year overall survival rates of 30–60% (9). However, these techniques are limited by the narrow field of view, suboptimal exposure, destroyed structures, and considerable surgical trauma. They are also associated with complications such as maxillary necrosis, palatal fistula, facial numbness, and a facial scar that seriously affect the patients’ quality of life (10).

With the development of nasal endoscopy-related surgical instruments and techniques in recent years, transnasal endoscopic surgery for recurrent NPC has gradually become a widely recognized safe and effective treatment modality (11–13). However, the great difficulty in dissecting middle- and advanced-stage lesions by nasal endoscopy, especially those invading the PPS and the internal carotid artery, and the risk of rupturing the internal carotid artery often result in incomplete tumor resection. Protection of the internal carotid artery during nasal endoscopic surgery is a key to surgical safety. For recurrent NPC, radiotherapy leads to local tissue necrosis and disordered scarring of the nasopharynx anatomical structures, making it difficult to recognize them and protect the internal carotid artery. A surgical plan for ruptured internal carotid artery bleeding should be established preoperatively. Patients at high risk of internal carotid artery rupture require surgery in a hybrid operating room. The internal carotid artery needs to be prepared for interventional embolization before the operation, and an imaging navigation system and ultrasonographic Doppler should be applied during the operation to help locate the internal carotid artery. Furthermore, such surgeries require highly experienced surgeons. These are objective conditions that prevent the widespread use of nasendoscopy surgery to treat recurrent and radiotherapy-insensitive NPCs. Studies have reported that rT2 stage lesions are more likely to invade the PPS, which is generally less accessible to nasendoscopy and has a low anatomical safety margin, resulting in a poor prognosis (14).

The above indicates that despite its advantages, endoscopic surgery alone has some limitations in treating recurrent and radiotherapy-insensitive NPCs. To address these issues, we designed a surgical strategy that combines nasendoscopy and a submandibular approach to resect NPCs that involve the PPS. Freeing the inferior pole of the parotid gland or dissecting the marginal manibular branch of the facial nerve to the carotid trunk during surgery can fully expose the PPS structure at the skull base and facilitate full dissection of the internal carotid artery PPS segment. Besides, this surgical approach facilitates the creation of a submental island perforator flap for nasopharynx wound repair and avoids life-threatening massive necrotic bleeding and denudation of the internal carotid artery. We uneventfully revealed and protected the glossopharyngeal, accessory, vagal, and hypoglossal nerves in the PPS and dissected the fat inside the carotid sheath and lymph nodes in all seven patients. Given the access to the PPS, it was easy to divert the submental perforator flap superiorly to the nasopharynx and perform surgical wound revision after tumor resection. All seven patients achieved primary healing of the incision and nasopharynx. While the patients had slight paresis of the facial nerve mandibular ramus postoperatively, all recovered completely within three months. The scar of the neck incision in the submandibular region is essentially invisible when the patient is in frontal view, achieving high patient cosmetic outcome satisfaction. The nasopharynx was repaired with a submental flap, leaving no exposed wounds. Postoperatively, there was only a short period of small crusting in the nasopharynx incision that had disappeared within 2–3 months. The patients had good nasal ventilation and no significant decrease in nasal function.

Our designed surgical approach has the following advantages: a) the surgery relies little on instruments and does not need the hybrid operating rooms or navigation equipment. It is relatively easy to dissect the internal carotid artery upward to the parapharyngeal segment through the neck. The surgical manipulations are performed under direct vision, and it is easy to locate the internal carotid artery using ultrasonographic Doppler. b) The submental flap protects the exposed internal carotid artery and nasopharynx wound after dissection and reduces the risk of patient death from massive hemorrhage due to internal carotid artery rupture. The submental flap had less local inflammatory exudation and scabbing and faster postoperative recovery than with temporalis flap repair. c) The retropharyngeal lymph nodes are the most common target NPC metastases (15). Studies have shown that surgical resection of metastatic retropharyngeal lymph nodes had superior outcomes to radiotherapy (16). The deep anatomical position of the retropharyngeal lymph nodes makes them difficult to localize, turning their complete resection while dissecting and protecting the parapharyngeal internal carotid artery difficult. Our designed surgical procedure involves dissection of the PPS and the retropharyngeal lymph nodes while removing the nasopharyngeal lesion. Intraoperatively, retropharyngeal lymph node dissection can be inspected thoroughly by endoscopy and palpation of the retropharyngeal space to detect any residual retropharyngeal space lymph nodes. d) The nasendoscopy field of view has the advantages of good mirror lighting conditions, close viewing, and a magnification effect, showing the anatomical structures clearly. Nasopharynx lesions were completely resected, relying on the advantages of the nasendoscope view. Robotic surgery has a good field of view and operational advantages for the resection of oropharyngeal masses, and robotic surgery can also play a good technical advantage in the management of tumors in the parapharyngeal space. However, the expensive equipment of robot limits the application and popularity of this surgery. In this contrast, our designed surgical approach does not rely on surgical equipment and has certain advantages and generalizability. There are indeed some inherent flaws. First, the limitations of the present study are the small sample size and the insufficient length of follow-up. Second, the new procedure designed in this study has certain requirements for surgeons’ experience and technique. Familiarity with the dissection of the lateral neck region and the cranial base with nasoendoscopy is essential for successful surgery. In the future, we will continue to collect accumulating case data on patients who are suitable for this surgical modality and continue to follow-up patients to obtain more long-term efficacy evaluation.

## Conclusions

En bloc resection of the tumor was obtained in the 7 patients without recurrence and serious complications during the follow-up. The findings of this cohort study suggest that, patients with rNPC and radiotherapy-insensitive types of nasopharyngeal carcinoma, the combined microinvasive trans-submandibular and nasendoscopy surgical approach may be considered as an surgical options. The results of this study provide an additional option for surgical treatment of NPC in the clinic. Although the sample size in our study was small, all patients achieved good treatment outcomes and had no serious complications, providing a reference basis for future clinical applications of the procedure.

## Data availability statement

The original contributions presented in the study are included in the article/supplementary material. Further inquiries can be directed to the corresponding authors.

## Ethics statement

The studies involving human participants were reviewed and approved by clinical research ethics committee of Beijing Tongren Hospital. The patients/participants provided their written informed consent to participate in this study. Written informed consent was obtained from the individual(s) for the publication of any potentially identifiable images or data included in this article.

## Author contributions

JF had full access to all of the data in the study and takes responsibility for the integrity of the data and the accuracy of the data analysis. Concept and design, JF, YZ, and QZ. Acquisition, analysis, or interpretation of data, LZ, CW, JC, ML, XS, and YY. Drafting of the manuscript, JF and YZ. Critical revision of the manuscript for important intellectual content, all authors. Statistical analysis, JZ, QS, and YZ. Administrative, technical, or material support, JC, LF, SH, LZ, and RW. Supervision, HM, LH, and CW. All authors contributed to the article and approved the submitted version.

## Funding

This work was supported by the Key R&D Program of China (No. 2020YFB1312805), Capital Health Research and Development of Special Fund (No.2022-1-2051), Priming Scientific Research Foundation for the Junior Researcher in Beijing Tongren Hospital, Capital Medical University (2021-YJJ-ZZL-018), National Natural Science Foundation of China (No. 82101187, 82002880), Beijing Municipal Administration of Hospitals Incubating Program(PX2021008), Beijing Hospitals Authority Youth Programme (QML20200205).

## Conflict of interest

The authors declare that the research was conducted in the absence of any commercial or financial relationships that could be construed as a potential conflict of interest.

## Publisher’s note

All claims expressed in this article are solely those of the authors and do not necessarily represent those of their affiliated organizations, or those of the publisher, the editors and the reviewers. Any product that may be evaluated in this article, or claim that may be made by its manufacturer, is not guaranteed or endorsed by the publisher.
